# User-centered design approach to visualize PROMs for molecular tumor boards

**DOI:** 10.1038/s41698-025-01061-x

**Published:** 2025-08-05

**Authors:** Sara Bäcker, Brita Sedlmayr, Miriam Goldammer, Katharina Schuler, Maria Zerlik, Cosima Strantz, Philipp Unberath, Linda Gräßel, Martin Sedlmayr, Markus Wolfien

**Affiliations:** 1 Institute for Medical Informatics and Biometry, Faculty of Medicine and University Hospital Carl Gustav Carus, TUD Dresden University of Technology, Dresden, Germany; 2 Medical Informatics, Friedrich-Alexander-Universität Erlangen-Nürnberg, Erlangen, Germany; 3 Medical Center for Information and Communication Technology, Universitätsklinikum Erlangen, Friedrich-Alexander-Universität Erlangen-Nürnberg, Erlangen, Germany; 4 SRH Fürth University of Applied Sciences, Fürth, Germany; 5 Department of Internal Medicine I, Hematology, Oncology and Stem Cell Transplantation, Medical Center - University of Freiburg, Faculty of Medicine, Freiburg, Germany; 6 Center for Scalable Data Analytics and Artificial Intelligence (ScaDS.AI), Dresden/Leipzig, Dresden, Germany

**Keywords:** Targeted therapies, Diagnosis, Quality of life, Therapeutics, Cancer genomics, Cancer screening, Cancer therapy

## Abstract

Integrating Patient-Reported Outcome Measures (PROMs) into Molecular Tumor Boards (MTBs) remains challenging due to the complexity of data visualization and integration into clinical workflows. This work, as part of the German PM4Onco project, aims to identify visualization requirements for PROMs and develop a prototype for PROMs integration in cBioPortal, facilitating broader application within oncology care. We employed a qualitative research approach, including developing personas for MTB stakeholders, conducting a literature-based requirements analysis, organizing a co-design workshop to create low-fidelity prototypes, and evaluating the highest-rated prototype variant through an online survey distributed to MTB physicians across Germany. Seven specialist groups were identified, with key needs including intuitive visualization, clear axis labeling, and longitudinal symptom tracking. The resulting mid-fidelity mockup incorporated PROMs data within cBioPortal’s timeline view and a detailed PROMs tab, featuring trend indicators, line graphs, and customizable health displays. Usability evaluation by MTB members yielded a SUS score of 67, indicating an initial indicator of acceptable usability, with suggestions for improvements like threshold scores and deeper clinical data integration. While PROMs offer critical patient insights, they remain underused in MTBs. Our early-stage prototype demonstrates potential for addressing this gap, with future work focusing on implementation, broader testing, and international validation.

## Introduction

There is a growing need to empower patients and enable their active participation in the clinical decision-making processes^1,2^. Incorporating patients’ perspectives on their health status into healthcare not only enhances patient-centered care but also improves the overall patient journey^3^. Patient-reported outcome measures (PROMs) serve to make the subjectively perceived health status by patients during or after treatment measurable and comparable. PROMs are essential for comprehensive clinical assessments and personalized treatment, offering insights into patients’ health status, quality of life, and treatment satisfaction^4^. Additionally, analyzing PROMs data within the patient journey can reveal patterns and trends that inform clinical practices and health policy. By integrating PROMs, healthcare providers can improve care quality and empower patients by recognizing their experiences and preferences^5^.

Despite their importance, PROMs scores are still rarely integrated directly into clinical decision-making^6,7^. In addition to the difficulty of integrating PROMs data into the patient’s electronic health record (EHR) and clinical information system (CIS), a major challenge is the lack of standardized graphical visualization formats that effectively convey the patient’s perspective within the clinician’s clinical workflow. Visualizing PROMs data becomes even more complex when integrating them with genomic data and clinical data in Molecular Tumor Boards (MTBs)^8^. An MTB consists of an interdisciplinary group of experts, such as bioinformaticians, molecular oncologists and molecular biologists, who review all available patient data, including the genomic profile of the tumor, to recommend the most appropriate treatment options for the individual patient. Effective visualizations of PROMs data alongside genomic and clinical data are crucial for a comprehensive understanding of the patient’s condition and for facilitating informed treatment decisions, since PROMs are independent of interpretation of a healthcare professional. Existing platforms such as the cBioPortal, MTBP, or MTPilot, are widely used to present clinical and molecular data that support MTBs in cancer diagnosis and treatment planning^5,8,9^. Integrating PROMs data into these platforms, alongside existing MTB data, could facilitate patient-centered treatment decisions.

Fostering this integration, our study on PROMs data visualizations is part of the Personalized Medicine for Oncology (PM4Onco) project^10^. PM4Onco aims to establish a permanent infrastructure for integrating and exchanging data from clinical and biomedical research while supporting physicians in preparing complex data for MTB discussions. The aim of this work is to identify the requirements for PROMs data visualizations that can enhance the MTB decision-making process in personalized medicine. We will develop low-fidelity prototypes for the cBioPortal platform and evaluate the preferred design variant with a survey among experiences stakeholders in personalized medicine and MTBs, ensuring a user-centered solution.

## Results

### Mapping MTB stakeholder needs through persona development

As a result of the persona development, the following target group descriptions were created (Table 1).

**Table 1:** Developed personas as description of the target group

Field	Pathology	Systems Medicine	Systems Biology	Molecular Biology	Hematology/ Oncology	Human Genetics	Bioinformatics
**Quote**	*“I wish for a visualization format for Patient-Reported Outcomes (PROs) that effectively and efficiently supports me in discussing a patient case within the setting of the Molecular Tumor Board. It should help me identify changes in a patient’s progress early on and enable improved and more targeted therapy decisions.”*						
**Fictitious name**	Dr. Gabriele Schmitt	Dr. Matthias Müller	Dr. Sabrina Weber	Dr. Malte Fischer	Dr. Stefan Becker	Dr. Sheng Wagner	Dr. Sebastian Meyer
**Position**	Medical specialist	Scientific employee	Scientific employee	Scientific employee	Medical specialist	Medical specialist	Scientific employee
**Institute/Clinic**	Institute of Pathology	Core Unit for Systems Medicine	Department for Systems Biology	Institute for Molecular Biology	Clinic for Hematology & Internal Oncology	Institute of Human Genetics	Institute for Bioinformatics
**Typical tasks**	Clinically-oncologically relevant molecular tumor diagnostics; differential diagnostic distinction; investigation of changes in patients’ genetic information; genetic characterization of tumors; support for diagnostic assessment and therapeutic decision-making; statements about disease progression	Connection of system-oriented information with clinical patient data; simulation of pathological processes and the underlying mechanisms on the computer to non-invasively test intervention options for their effectiveness	Use of computer algorithms and mathematical models to collect, process, and analyze large amounts of clinical and biological data, and to build a profound (mechanistic) understanding of severe diseases; the models developed in this way can assist physicians in making faster decisions about the optimal (and personalized) patient treatment	Conducting molecular biological analyses; performing DNA sequencing; collecting and evaluating data; interpretation of research findings	Prevention and research of tumors; confirmation of diagnosis/monitoring of cancer/proof of treatment success via biopsy, imaging techniques, colonoscopy, histology and cytology, tumor markers; counseling of patients and relatives regarding possible measures	Differential diagnostic clarification, individual risk assessment, and human genetic counseling for genetically predisposed or associated tumor disposition; conducting, evaluating, and reporting on molecular genetic investigations; conducting analyses and reporting on the detection of somatic mutations in various tissues	Simulation of biochemical processes and biological data, such as the structure of DNA molecules or proteins on the screen; handling various bioinformatics databases; visualization of the structure of specific molecules or other compounds and application of these models in further procedures; development of software tools for data preparation, evaluation, and analysis
**Age**	50–59 years	30–40 years	30–40 years	30–40 years	50–59 years	50–59 years	30–45 years
**Language**	German						
**Professional experience**	High						
**MTB experience**	High, frequent participation						
**cBioPortal experience**	Medium-High						
**PROMs experience**	None	None	None	None	Medium-High	None	None
**Goals**	Primary:						
	. Improved treatment decisions by incorporating the patient’s perspective						
	. Improvement in the quality of care						
	Secondary:						
	. Support for shared decision-making or improved communication when discussing treatment results/outcomes with the patient (downstream)						
	. Use of aggregated PROMs scores in clinical trials to inform patients about the benefits and risks of treatment						
**Fears**	. Poor integration in cBioPortal, cumbersome access						
	. Poorly designed user interface (confusing, incomprehensible designations, etc.)						
	. Incomplete or outdated information of PROMs						

### Identification of clinically relevant visualization requirements for PROMs data

From the 18 included articles, a detailed table was created listing specific requirements for PROMs data visualizations tailored to MTBs, along with solution strategies and examples. This table provides a structured overview of the essential elements required for effective integration of PROMs scores into clinical practice (see online supplementary material part A). Key requirements include the need for PROMs data visualizations to be intuitive, efficient, and compatible with the demands of a clinical setting^11,12^. Visualizations should be easily accessible for individual patient views and applicable for users with varying levels of experience. To minimize cognitive load, the visual complexity should be kept low, allowing quick interpretation during a busy clinical workflow.

**Fig. 1:**
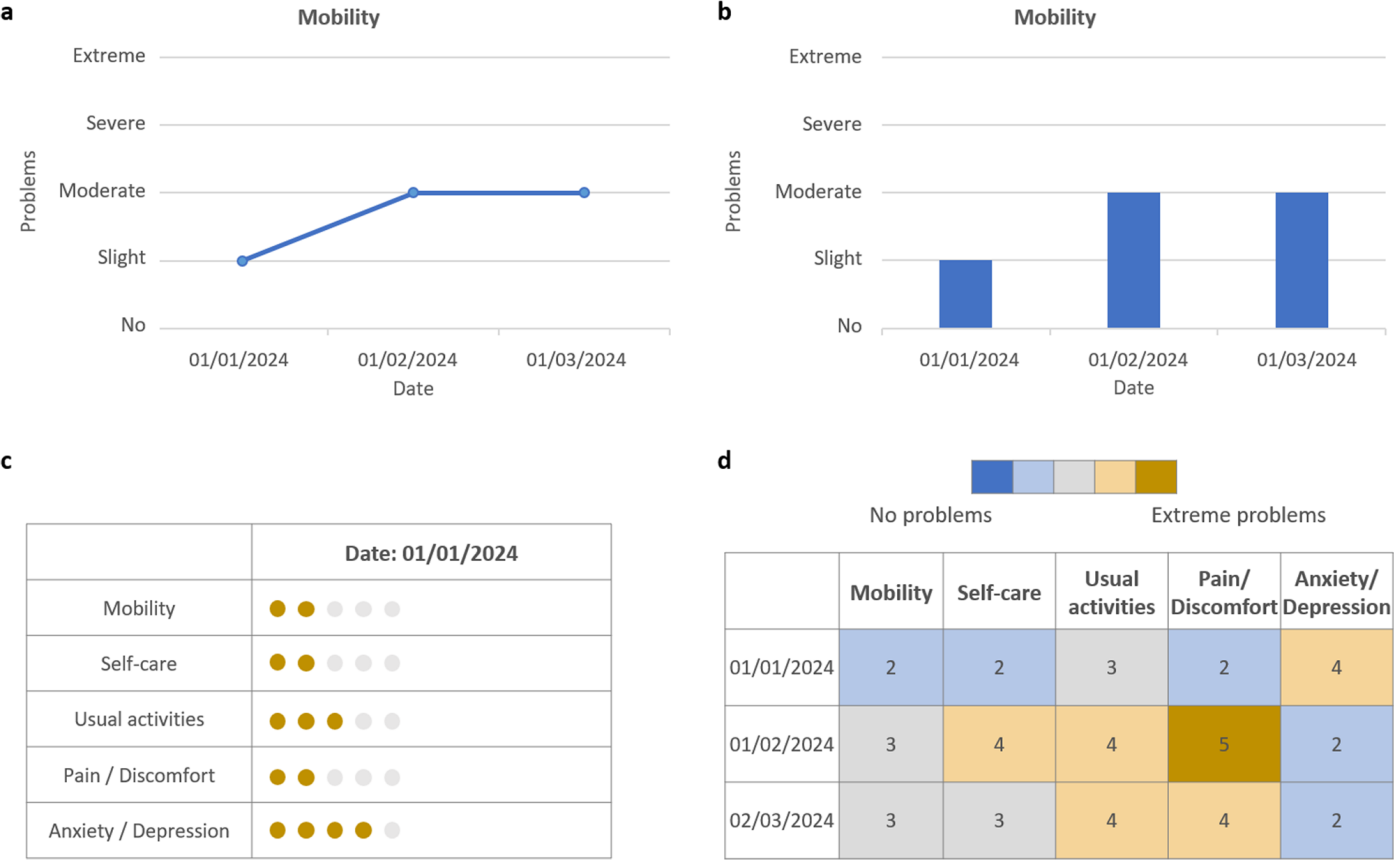
Examples of visualization strategies for patient-reported outcome measures (PROMs). Suitable graphic visualization formats for PROMs data based on^18^, including **a** line graph, **b** bar chart, **c** icon array, and **d** heat map.

For an intuitive use, PROM visualizations should aim for simplicity and clarity, with consistent scoring direction (e.g., higher scores indicating improvement) and a limited number of displayed metrics. Guided interpretation is essential to save clinician’s time and focus, achievable through graphical cues such as color coding, highlighting critical scores, and marking areas requiring clinical attention^13,14^. A longitudinal view is essential to monitor symptom progression and pharmacovigilance in relation to clinical interventions. To aid interpretation, visualization should include all necessary contextual details, such as clear labeling (e.g., mild/moderate/severe), directional indicators, and score thresholds or reference data for comparison^14^. The visualizations need to be adaptable to different target groups through personalized and interactive views with varying levels of detail. Identified visualization formats include bar charts, line graphs, pie charts, funnel plots, heat maps, icon arrays and pictographs. The four most frequently used formats applicable to the specified use case of individual-level PROMs data visualization are shown in Fig. 1. Although no single format was universally preferred, line graphs and bar graphs emerged as the most intuitive and familiar, reducing potential misinterpretation. In contrast, pictographs were reported as the least useful for clinical interpretation^11,13^.

### Prototype development through a user-centered co-design workshop

**Fig. 2:**
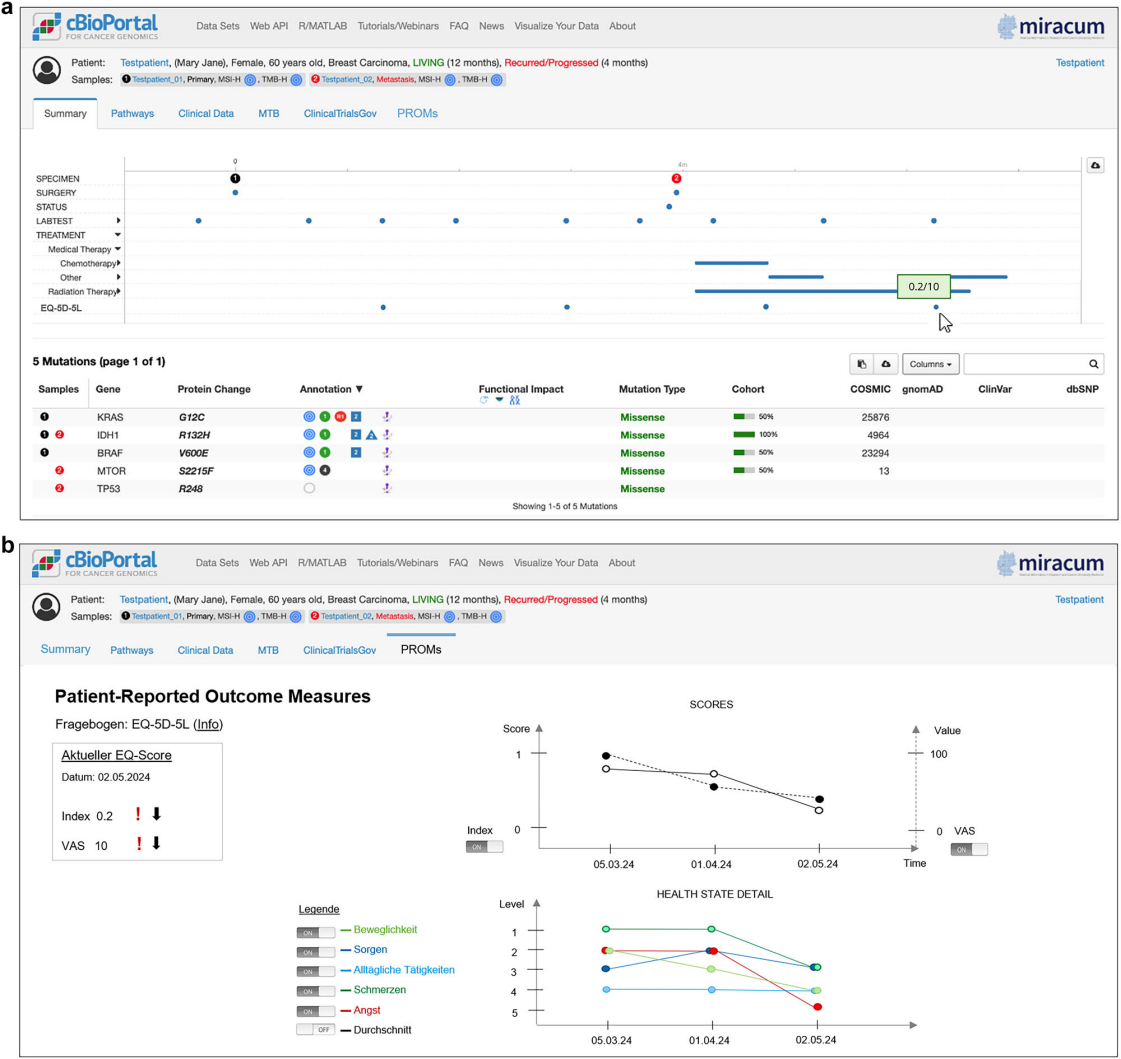
Mockup for the visualization of PROMs in cBioPortal on an individual level. **a** Summary tab, **b** PROMs tab. General cBioPortal elements were provided by Strantz et al.^10^ and have been adapted for our PROMs tab. Screenshot of the MTB-cBioPortal interface, an open-source tool licensed under the GNU AGPL v3 (Source: https://github.com/PM4Onco/MTB-cBioPortal).

The co-design workshop included 31 scientists, researchers, and physicians from nine different locations. The workshop resulted in four low-fidelity prototypes for integrating PROMs data into cBioPortal (see online supplementary material part B). The visualization that the participants considered most appropriate to meet the needs identified in the previous requirements analyses was selected and transformed into a digitized mockup, following exactly the design proposed in the workshop. Figure 2 shows the digitized mockup.

In the preferred design, PROMs data is integrated into the “summary” tab of the patient view page (Fig. 2a) in cBioPortal by displaying the scores as points in the timeline. This allows MTB members to correlate PROMs data with the specific clinical events, such as surgeries, laboratory tests, and therapies. Each PROM result at a given time point is viewable through a mouse-over function for quick reference. Additionally, a new “PROMs” tab (Fig. 2b) provides more detailed information. The left side of this tab displays the current EQ-5D-5L index score and VAS value with trend arrows indicating changes. The top-right section shows these values over time in a line diagram, while the lower section presents detailed health levels in another line chart. Users can customize the view by toggling specific parameters on and off, enhancing the tool’s flexibility for varied clinical needs.

### Usability evaluation of the mockup for the visualization of PROMs

A total of 35 individuals were invited to participate in the survey via project mailing lists and direct outreach, of which 10 responded. The participants were from at least four institutions across Germany, with most respondents working in hematology/oncology, participating in an MTB, and reporting low experience with cBioPortal.

**Fig. 3:**
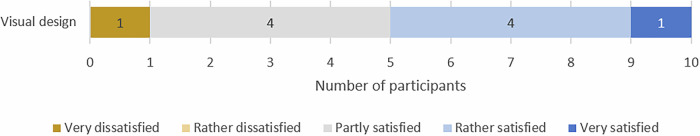
Participant satisfaction ratings regarding the visual design of the PROM mock-up. Horizontal stacked bar chart showing the distribution of responses (n = 10) across five satisfaction levels, ranging from very dissatisfied to very satisfied.

The SUS score for the evaluated mockup for PROMs data visualization was 67, which is interpreted as “borderline to good”^15^. This indicates a satisfactory level of usability with room for improvement. Most respondents envisioned using the application regularly, found it well-integrated, and felt it would be easy to learn without requiring technical support. Overall, the mockup was perceived as intuitive and easy to use. Regarding overall satisfaction with the visual design, most respondents were at least partly satisfied (Fig. 3), indicating potential areas for enhancement. Interactive elements such as on/off switches and mouse-over features received positive feedback for usability.

**Fig. 4:**
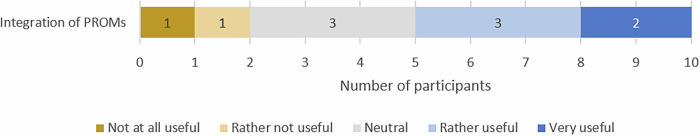
Perceived usefulness of integrated PROMs into the MTB workflow. Horizontal stacked bar chart illustrating participant responses (n = 10) on a 5-point Likert scale ranging from not at all useful to very useful.

The majority of respondents felt the mockup was sufficiently comprehensive, yet provided valuable suggestions for improvements. Key recommendations included adding warning signals, comparative scales, and enhanced integration with molecular and clinical data. Opinions on the benefits of integrating and visualizing PROMs data for MTBs in cBioPortal were somewhat divided, with most respondents rating this aspect as neutral or rather useful (Fig. 4). All questionnaire items with the respective answers can be found in the supplementary material part C.

These findings provide a strong foundation for developing PROMs data visualizations tailored to MTB needs, underscoring the importance of intuitive, effective software tools that support accurate data interpretation.

## Discussion

The visualization of PROMs data for MTBs must address a diverse group of specialists including molecular pathologists, systems physicians, biologists, oncologists, geneticists, and bioinformaticians. Since the target group is limited to the MTB members and the preparation team, other potential stakeholders, such as nursing staff, general practitioners, and patients are not included at this stage. Notably, no established personas for MTBs exist in the current literature, highlighting the novelty and relevance of this work. These personas, while not fully developed through empirical data, were supplemented by specific project assumptions. However, they proved to be effective communication tools for capturing and conveying the varied needs of MTB stakeholders. Although this target group is specialized, there is considerable variation in digital literacy and familiarity with systems like cBioPortal. A well-designed, intuitive visualization can significantly improve system adoption among these users, supporting efficient decision-making and reducing the time required for data interpretation. Overall, these personas provide valuable insights for tailoring the visualizations of PROMs data to meet the specific needs of MTBs, reinforcing the importance of usability in clinical tools to support personalized oncology care.

Graphical visualizations of PROMs data have significant potential to support patient-centered care^16,17^, though several key factors such as intuitiveness and efficiency need to be considered. While the literature has not identified a universally preferred format, line and bar graphs are commonly used for reasons of clarity and ease of interpretation^18^.

Achieving a balance between clarity and information depth is essential for ensuring usability^18^. Although dynamic dashboards with customizable views could meet diverse user needs, their technical implementation should remain cost-effective to ensure feasibility within clinical settings. The PM4Onco project’s scientific focus introduces specific demands for detailed analyses, including statistical details (e.g., *p*-values, confidence intervals). However, MTB patients represent a unique group with advanced or rare tumors, lacking established threshold values and representative comparison cohorts. It remains to be investigated whether comparing PROMs scores to similar patients or healthy individuals provides the most meaningful insights for MTB use. Alarm mechanisms for abnormal PROMs scores are likely unnecessary in the MTB context, as the MTB primarily focuses on providing treatment recommendations rather than ongoing patient management.

Integrating PROMs scores with clinical data, such as therapy timelines and medication changes, could provide a holistic view of the patient’s condition without interpretation by health care professionals, thus enhancing patient-centered decision-making and therapy monitoring. Yet, practical limitations in data availability and integration across cBioPortal locations restrict the feasibility of such comprehensive views. The requirements analysis was limited by the inclusion of only 18 articles in the analysis and that no systematic literature review was conducted. Nevertheless, the comprehensive review by Albers et al.^18^ and its included articles provided a strong foundation for identifying key requirements for PROMs data visualizations in MTBs.

The co-design workshop effectively promoted a user-centered design approach, ensuring that the developed solutions closely aligned with the actual needs and preferences of clinicians. This alignment is essential for fostering higher satisfaction and acceptance of the final visualizations. By incorporating diverse perspectives, the workshop encouraged creativity and innovation, while the collaborative environment fostered teamwork and uncovered novel insights. However, several challenges emerged during the workshop. It required careful planning and skilled moderation to manage group dynamics effectively. The diversity of participants was also crucial, as unrepresentative user groups could result in incomplete insights. Additionally, dominant voices sometimes overshadowed quieter participants, potentially skewing the overall understanding of user needs. Alternative methods, such as interviews, surveys, and usability testing, could provide additional insights, but were not employed due to time constraints and their comparatively lower level of collaborative engagement. The co-design workshop provided immediate feedback and fostered direct engagement, making it a particularly effective choice for quickly iterating design ideas.

The workshop generated four distinct design variants, each with strengths and limitations. An important observation emerged regarding the interpretation of graphic formats across user backgrounds. While some participants found certain graphics intuitive, others perceived the same graphics as complex and challenging. This variation in interpretation highlights the need to consider diverse user perspectives when making design decisions to ensure broad accessibility. The final workshop outcomes aligned well with the initial requirements analysis. Directional consistency was maintained across graphs, complexity was kept manageable, line graphs were used which were generally well received, and detailed score analysis was supported. This alignment validates the effectiveness of the co-design approach. Nonetheless, the time-limited setting resulted in a low-fidelity prototype, highlighting the need for further enhancement. Specific areas of improvement include clearer axis labeling, detailed explanations of score meanings, and the inclusion of score thresholds. Additionally, the technical feasibility of the mock-up will need to be assessed in the subsequent stages of its implementation in cBioPortal. In summary, the workshop successfully highlighted user preferences and provided a solid foundation for the next steps in prototype refinement and implementation, ensuring that the design remains user-centered and technically feasible.

The usability evaluation of the mid-fidelity mockup reveals both promise and areas for improvement in visualizing PROMs data. The survey was conducted as an explorative assessment to gather initial impressions of usability and perceived usefulness among German MTB professionals. Given the context-specific nature of the mockup, which reflects workflows in the German healthcare system, the study was not designed for international generalizability or statistical representativeness. The SUS score of 67 is of limited significance since only 10 physicians participated in the survey. The SUS score of 67 should be interpreted as an initial usability indicator, reflecting early impressions from a limited but highly experienced sample. However, it is challenging to involve more participants in the constrained time available in clinical settings^17^, therefore the limited number of participants was considered appropriate in this early development stage. The divided opinions on the graphical design of the mockup suggest that further refinement is necessary to fully address the complex information needs of MTB physicians. The current state of development reflects a mid-fidelity mockup designed to gather initial user feedback and define design requirements. The results obtained are actively used to prepare the technical implementation of the visualization components within cBioPortal. This includes leveraging existing APIs and data models (e.g., the international communication standard HL7 FHIR), to ensure interoperability with clinical data systems^19^. This staged approach allows us to refine both the visual design and backend logic with real-world constraints in mind, rather than over-engineering without clinical input.

PROMs data provides valuable insights into the patient’s subjective experience, capturing aspects of health and quality of life that are difficult to observe in clinical settings - standardized and free of interpretation. This holistic understanding of symptoms, functional status, and well-being is essential for informed, patient-centered decisions^20^. While PROMs scores may not significantly impact every MTB decision, they contribute substantially to the overall patient journey by offering insights that enhance personalized care. Moreover, we recognize the future potential of PROMs in supporting comparative patient analyses^21^. In situations where patients present with similar molecular and clinical characteristics, PROMs data could allow clinicians to compare individual symptom trajectories or quality-of-life outcomes across different treatment paths. This could add an additional layer of data-driven insight to individual therapy selection and further reinforce the role of PROMs in precision oncology.

This user-centered design approach applied in this study lays a foundation for incorporating PROMs data into clinical workflows. Not only in MTBs, but also for organ boards and in other fields, such as cardiology, neurology, and rare diseases. Currently, few software tools support the integration of PROMs data with molecular and clinical information^22^. Addressing the technical challenges of incorporating PROMs data into EHRs and integrating them with comprehensive visualizations remains critical. Overcoming these challenges will be key to realizing the full potential of PROMs in various areas of medical research and care.

Taken together, the described user-centered design approach proved to be a valuable method for early-stage development, effectively gathering diverse user perspectives and generating rapid, low-fidelity visualization solutions. While managing group dynamics and ensuring representative participation in the co-design workshop posed challenges, the benefits of rapid ideation and collaborative engagement outweigh these limitations. This work provides essential insights into the requirements and user needs for PROMs data visualizations in MTBs, particularly emphasizing the importance of clarity, usability, and tailored data presentation. Based on the usability evaluation, the developed mid-fidelity mockup will be further refined and implemented in cBioPortal, with plans for iterative testing and feedback collection to ensure alignment with clinical workflows. Future work will address technical integration challenges and explore scalable solutions to incorporate PROMs into EHRs, ultimately enhancing patient outcomes through improved data accessibility and usability.

## Methods

A qualitative research approach was employed to investigate concepts, experiences, and perspectives related to the visualization of PROMs data. Key stakeholders for the visualization of PROMs data within MTBs were identified, followed by a requirements analysis to determine specific visualization needs. A co-design workshop was conducted to collaboratively develop low-fidelity prototypes, which were subsequently evaluated to assess usability and effectiveness.

### Development of personas

To identify target groups and their specific characteristics for the design of PROMs data visualizations, we developed personas, fictional characters representing different user types with their individual needs, experiences, and goals^23^. These personas were based on roles that actively participate in MTB case discussions, which typically include medical oncologists, pathologists, molecular biologists, geneticists, and bioinformaticians^24,25^ Roles such as nurses and data managers, while essential to patient care and data handling, are not regular participants in MTB sessions within the German clinical context and were therefore not included in the persona development. For each target group, characteristics were gathered through an analysis of existing literature, including peer-reviewed studies, official statistics, and publicly available information from reputable organizations. This approach ensured a representative understanding of each group’s needs. Key characteristics were categorized into areas of activity/relevant tasks, average age, gender distribution, professional experience, fears/concerns, desires, and expectations. Additional categories, such as goals, prior experience with cBioPortal, and familiarity with PROMs, were included as assumptions specific to the PM4Onco project. The collected data were organized in tabular form to effectively inform the design process.

### Requirements analysis

The requirements analysis was conducted using a review-based approach. Initially, a comprehensive and current systematic review by Albers et al.^18^ on PROMs data visualizations with a focus on oncology provided a strong foundation. This review and its included articles, along with additional articles from a previous search on visualization strategies^26^, formed the basis of our analysis.

Studies that specifically addressed the visualization of PROMs data representing an individual patient view from the clinician’s perspective were included. The objective was to identify clinician-centered requirements for PROMs data visualization solution. The patient’s perspective was not considered, but planned to incorporate it at a later stage. After abstract screening of 26 articles, 18 were deemed relevant for full-text analysis.

Findings from these articles were extracted and organized into a three-tiered requirements table, which includes: general requirements for PROMs data visualizations, recommended strategies for visualization solutions, and specific examples for visualization formats.

### Co-design workshop

The primary objective of the workshop was to collaboratively develop an initial prototype for PROMs data visualizations within cBioPortal, based on requirements identified through prior literature and requirement analysis. The workshop was planned for 30 participants, including developers (e.g., medical informaticians, bioinformaticians, systems biologists) and MTB members, each contributing their expertise to foster a comprehensive design approach.

Prior to the workshop, participants received introductory material outlining the workshop’s background and goals, the target groups (represented by the developed personas), minimum visualization requirements, the EQ-5D-5L questionnaire as a specific PROM example, and screenshots of cBioPortal’s existing interface. This preparation ensured that participants were well-informed. The standardized questionnaire EQ-5D-5L to assess the patient’s quality of life, was chosen to be visualized in cBioPortal. It consists of two parts: the EQ-5D descriptive system and the EQ VAS. The EQ-5D descriptive system comprises five areas: Mobility, self-care, usual activities, pain/discomfort, and anxiety/depression. Each domain has five health levels, from “no problems” to “extreme problems”. Depending on the level, each dimension is assigned a number, resulting in a 5-digit number combination, which can be converted into an index score ranging from 0 to 1. The EQ VAS assesses the patient’s general state of health, with 100 representing the best and 0 the worst imaginable state of health.

Held in July 2024 as an in-person event in Dresden, Germany, the workshop began with an introductory talk on user-centered design. Participants were then divided into four design groups, each moderated by a usability expert. Groups were assigned two tasks: a) to determine where PROMs scores should be integrated within cBioPortal, and b) to create a low-fidelity prototype (paper based) of the PROMs data visualizations. To assist with the design process, each group was provided with a handout detailing all relevant information, an overview of design patterns, and materials for paper-based prototyping.

After completing the tasks, each group presented their designs in a plenary session. Participants then voted on the four different designs, selecting one for further development into a digitized mid-fidelity mockup, which was created using Microsoft PowerPoint to simulate the proposed user interface and interaction patterns. This mockup will be evaluated by MTB physicians to ensure it meets clinical user needs.

### Evaluation of the mid-fidelity mockup

For evaluation, an online survey was created using SoSci Survey^27^, featuring an embedded demonstration video of the mockup. The survey items were based on standardized usability questionnaires, such as the System Usability Scale (SUS) and supplemented with specific questions tailored to the study’s needs. Prior to distribution, the survey and the demonstration video were pre-tested by five experts from the Centre for Medical Informatics Dresden. Based on their feedback on clarity and relevance, adjustments were made.

The survey evaluated various aspects of the mockup, including surface design (e.g., clarity, aesthetics), applicability, user-friendliness (e.g., accessibility), and comprehensiveness. The questionnaire was distributed in September 2024 via the PM4Onco project mailing list and shared with 35 MTB physicians across multiple German institutions. The survey was open for three weeks, allowing participants to provide quantitative ratings and qualitative feedback on the mockup. A power analysis was not conducted, as the study was not designed to test hypotheses with statistical significance but rather to explore initial user impressions and identify usability considerations.

## Supplementary information

Supplementary material

## Data Availability

All data used were either publicly available, as indicated as reference, or are provided within this article and its supplementary information.
