# Supplementary material for: User-centered design approach to visualize PROMs for molecular tumor boards
Source: NPJ Precis Oncol. 2025 Aug 5;9:273. doi: 10.1038/s41698-025-01061-x (PMC12325938; doi:10.1038/s41698-025-01061-x)
Supplement: Supplementary file 1 — Supplementary material [file 41698_2025_1061_MOESM1_ESM.docx]

Supplementary material

# Requirements on PROMs data visualizations

Table 1: Requirements on PROMs data visualizations, including general requirements for PROMs data visualizations, recommended strategies for visualization solutions, and specific examples for visualization formats

| **Requirement** | **Strategy** | **Examples** |
| --- | --- | --- |
| Intuitive interpretation at a glance [1] | Simplistic and easy to read [2], comprehensible [3] | score directionality/ directional consistency (higher = better) [1] [2] [4] [5] [6] [7] [8] |
|  |  | reduce the number of metrics presented [1] [2] [9]  Keep it simple: limit number of symptoms, ask patients to prioritize [10]  maximum number of bars presented within a bar graph should be six, while the maximum number of lines within a line graph should be four [2] |
|  |  | Single page [11], minimizing page counts [2] |
|  |  | Heatmaps and color coding [1] [2] [12] |
|  |  | Emphasize trends [1] [13] |
|  |  | Best graph format?  Line [1] [2] [3] [4] [5] [14] [15]  Bar [1] [2] [14]  Table [15], Pie [4] [8] [16]  Pictographs are the worst [1] [17] [18]  With color-coding [12] [13] [15]  All the same [1] [12] [17] |
| Interpret PROMs correctly [1] | Provide all necessary information [1] and detail, including instructions where they will be needed [2], comprehensible [3] | axis labeling/ clear labeling [1] [4] [11] |

|  |  | detailed information on the meaning of high and low scores [1]  include descriptive labels (e.g., mild/moderate/severe) alongside the chart, assuming data to support the use of these thresholds are available [2]  one suggestion to avoid potential confusion is to provide a label to denote ‘better’ alongside the chart to indicate the directionality of the PROs or use colored arrows: green for better scores and red arrows for worse scores [2] |
| --- | --- | --- |
|  |  | brief definitions to understand what the PROMs scores represent [1] |
|  |  | detailed explanatory legend of the meaning and interpretation of colors and scores [1] |
|  |  | accompany more complex displays like funnel plots with a detailed interpretation [1] [2]  information buttons on “how to interpret this chart” [11]  annotations such as indications of score meaning and clinical significance may assist with clinical interpretation [8]  written explanations are particularly valuable for complex graphical displays [2] |
|  |  | corresponding summary table [13]  provision of a written explanation of the PRO score alongside the graph has also been recommended to assist with interpretation [2] |
| Guiding interpretation [1] [11] to save time and concentration for actual decisions to be made on the data | clinically applicable [3], emphasize implications of the findings [3] | display of score thresholds [1] [6] [7] [6]  highlighting scores requiring clinical attention [4] [15] [6]  visual cues to easily differentiate mild symptoms from moderate or severe [13] |
|  |  | warnings if scores change over time [1] |
|  |  | scores from reference populations to compare individual scores to [1] [2] [15]  reference population: similar patients [2] [12] [14] or healthy individuals [2] [12]  illustrate PROMs for both individual patients and panels of patients [11]  viewing key comparisons, such as aggregate PROs over time (i.e., pre-op versus post-op) [11] |
|  | trustworthy Data | Consider sample size [3] |
|  |  | Consider patient parameters that are relevant for accurate interpretation, e.g., smoking status, comorbidities, opioid use [3] |
| Support deeper (scientific) data analysis | enough details [18], clinically applicable [3] | Call for more statistical details:  representation of data variability (e.g., confidence intervals, error bars) [18]  introduction of funnel plots, heat maps, and icon arrays [1]  Qualitative results support that clinicians value confidence intervals, norms, and p values [15]  preference towards the inclusion of statistical details for PROs data [2]  there is a move away from reporting the p value alone to illustrate statistical significance, and instead the use of CIs is encouraged [2]  clinically important difference should also be included within the graphical representation of the PROs results, where appropriate [2] |
|  |  | Nevertheless, added value of more statistical details is questioned:  adding asterisks for clinical significance and confidence limits around scores did not contribute to a better interpretation accuracy in clinicians [1] [4] [8]  Clinicians both endorsed and objected that p-values, confidence intervals, and normed scores could contribute to their interpretation accuracy of aggregated PROMs data [1] [8]  while the addition of error bars to the graphs was generally considered to provide more information and was preferred by some, the bars made the line graph ‘‘too visually complex’’ for others [3] |
|  |  | Avoid replacing numbers with pictographs [18] |
| Monitor patient and therapy [1] [13] | track patients’ symptom experiences [1] / help understand how symptoms have changed over time [13] | Longitudinal view of patients [1] [3] [9] [15] comparison with previous personal results [13] [14]  ability to track symptoms over time [10]  long-term collection of PROs is necessary to achieve value [11] |
|  |  | Show PROMs together with clinical actions taken in the encounter [2]  correlated PROMs longitudinally to the patient’s medication regimen [9]  included laboratory values relevant for care [9]  dashboard as potential method for aggregating data from various sources [5]  Add information, e.g. explain why medication was discontinued [5]  link number scale to narrative [10]  ability to track symptoms over time and effect of intervention [10] |
| Support physicians/staff in decision making | anticipate which patients may need a more focused symptom assessment at their next appointment [13] | a visual flag to identify patients struggling with a high number of symptoms or concerns at their last visit [13] |
|  | receive an alert when scores indicated a clinically relevant deterioration in functioning or increase in symptoms [12] |  |
| Different intentions for visualizing PROMs/ different target groups [1] | Personalized/ Interactive views for deeper analysis [1] | design multiple formats per target group, as no ‘one-size-fits-all’ solution in graphic visualization exists [1]  two different dashboards would be more effective— one for physicians with more detailed information and another for patients that was more limited [5] |
|  |  | Dynamic dashboard that can be toggled to other formats and include error bars that frame comparison group scores [12] |
|  |  | Customize dashboard to own needs [5]  predefined views for most users, interactive design to dynamically explore the data for "power users" [11]  designing PRO Dashboards that enable users to tailor views with outcomes they care about most [11] |
|  |  | interactive dashboards were better suited for deeper analytics that support QI tasks and “talk with colleagues” to examine unusual patient cases [11] |
|  | support QI activities [11] | analyzing PROs of their own patients to reflect on their practice [11]  clinic-level and institution-level PROs aggregated across patients, better support QI activities [11]  in an exploratory study, participants warned that providing comparison data can have   unintended consequences, such as negative comparisons leading to reputational damage when the health-service or healthcare professional is reported to be lesser performing in their PROs results [2] |
| Easy to use in busy clinical routine / efficient [10] [17] | Easy to find PROMs-visualizations for one specific patient | a list of their patients from which each individual dashboard could be quickly accessed [13]  presentation of patient-level and provider-level PROs appear to provide the best support for patient care activities [11] |
|  | usable across a range of users (users that are more or less open to new tools); usable by individuals with varied workflows and experience [11] | Concern: utilization of HRQOL dashboards in their clinic could adversely impact the efficiency of their clinics and inhibit patient throughput [17]  concern: information overload [10]  concern: possibility of identifying problems without access to intervention [10] |

# Low-fidelity prototypes (paper-based) developed in the co-design workshop

**
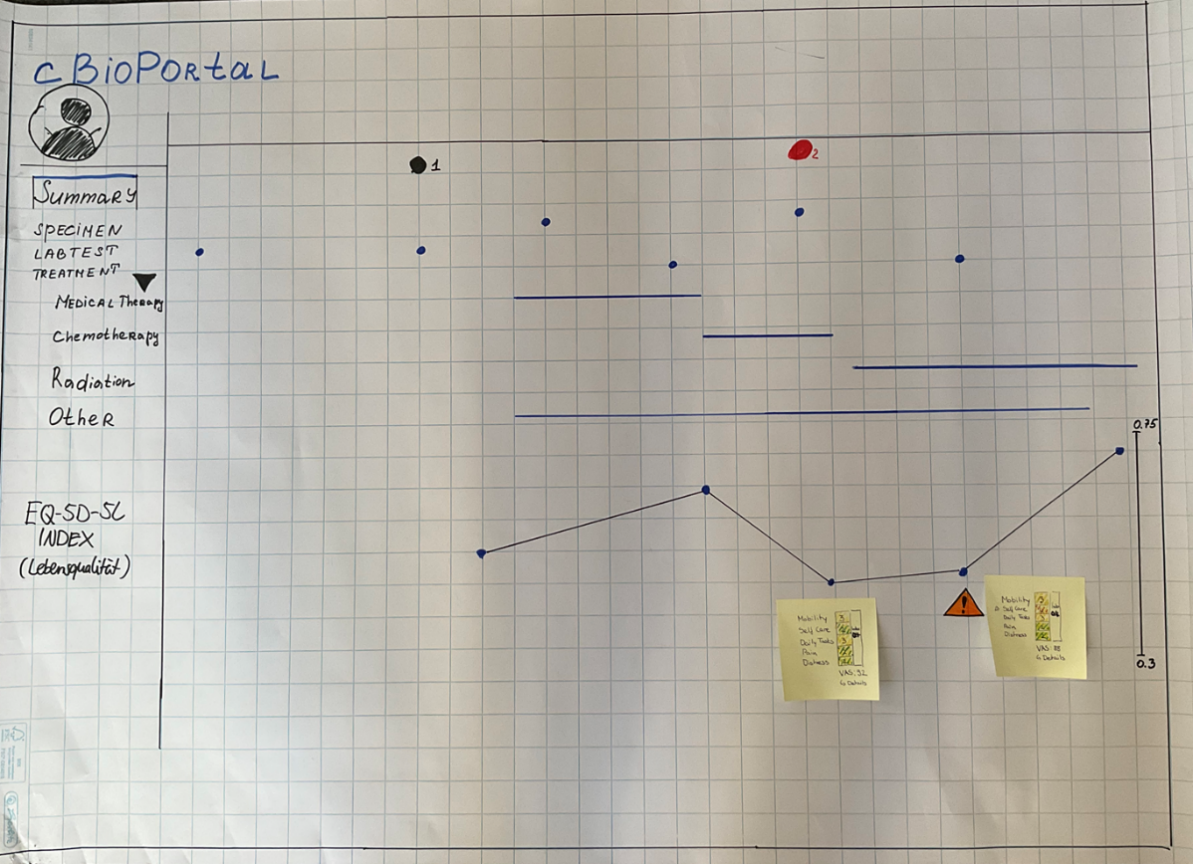
**

Figure 1: Prototype of design group 1, summary tab


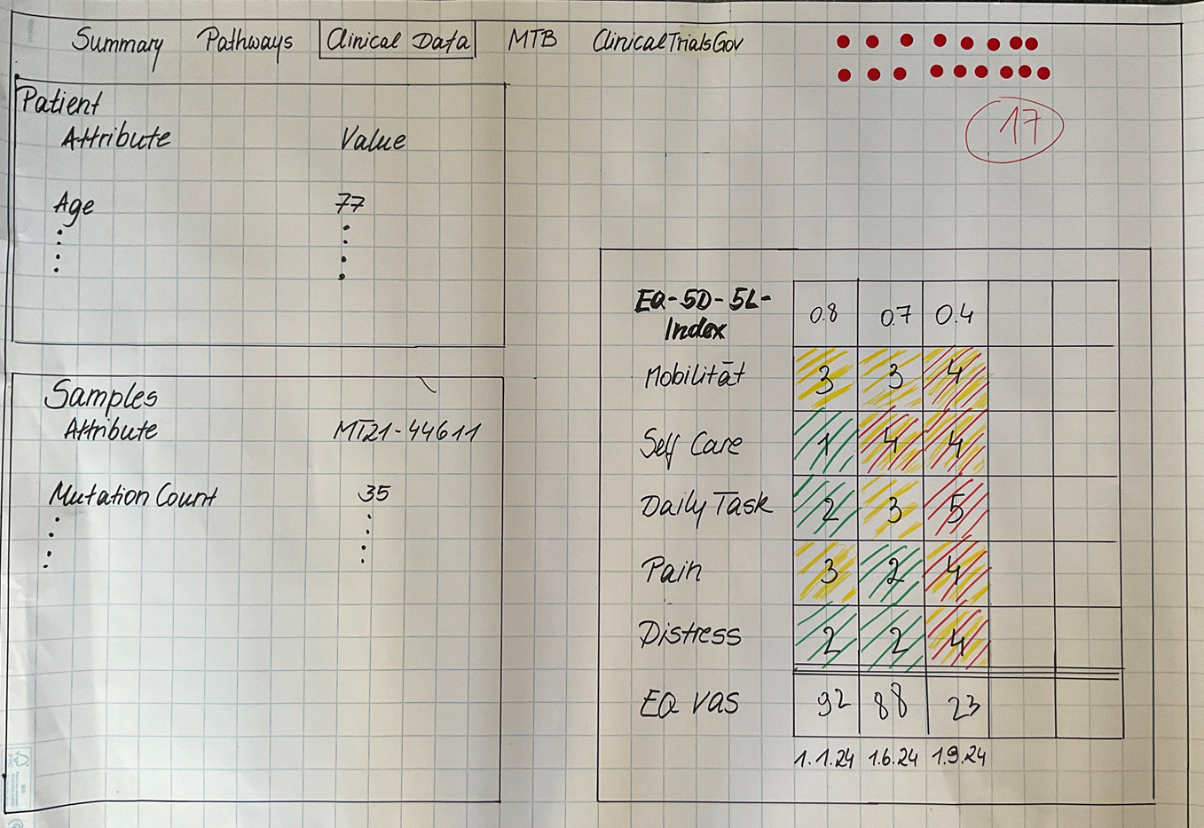


Figure 2: Prototype of design group 1, clinical data tab

**
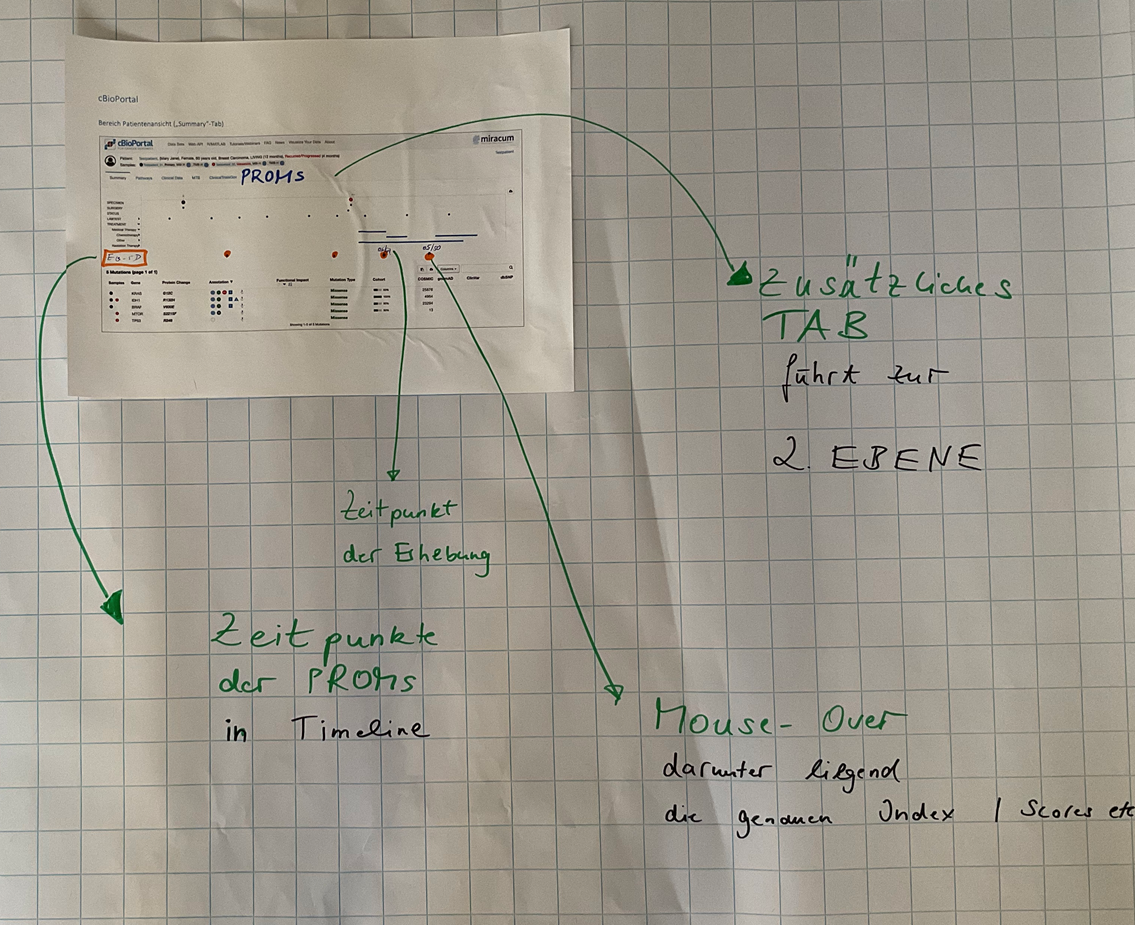
**

Figure 3: Prototype of design group 2, summary tab

**
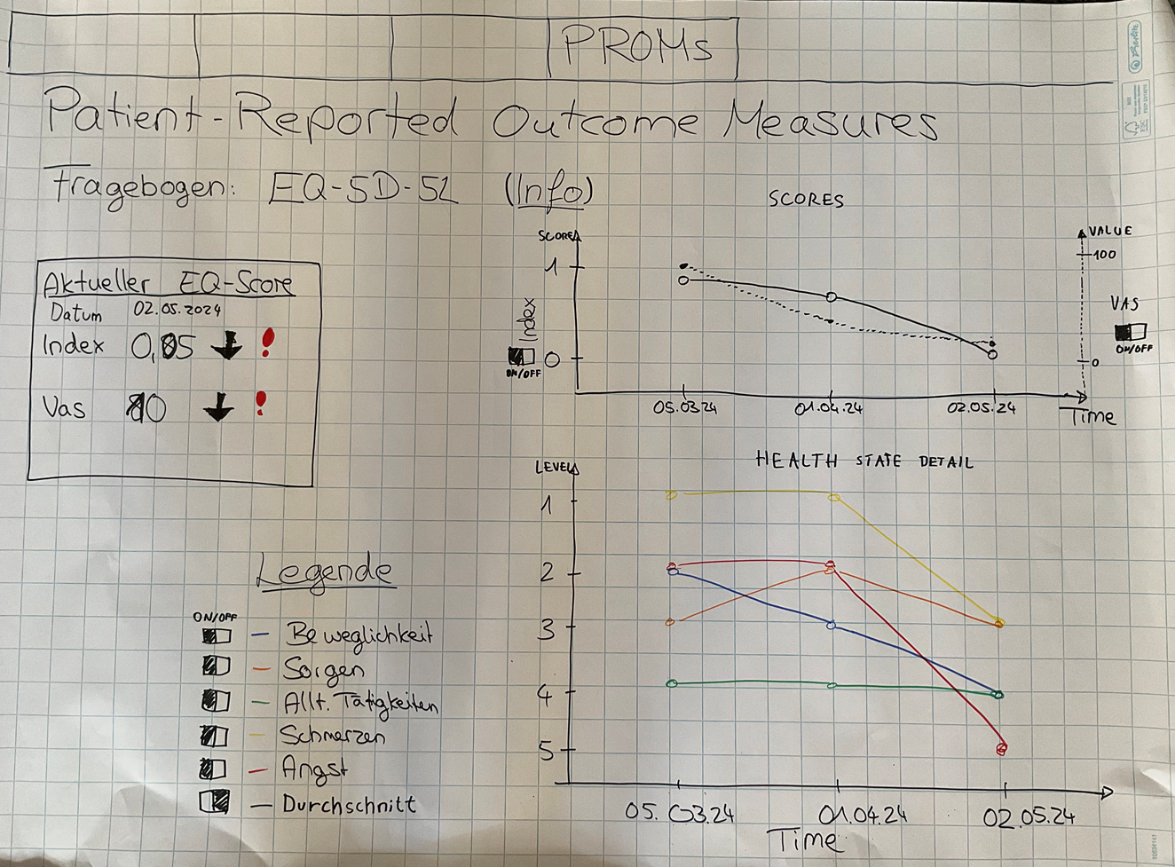
**

Figure 4: Prototype of design group 2, PROMs tab

**
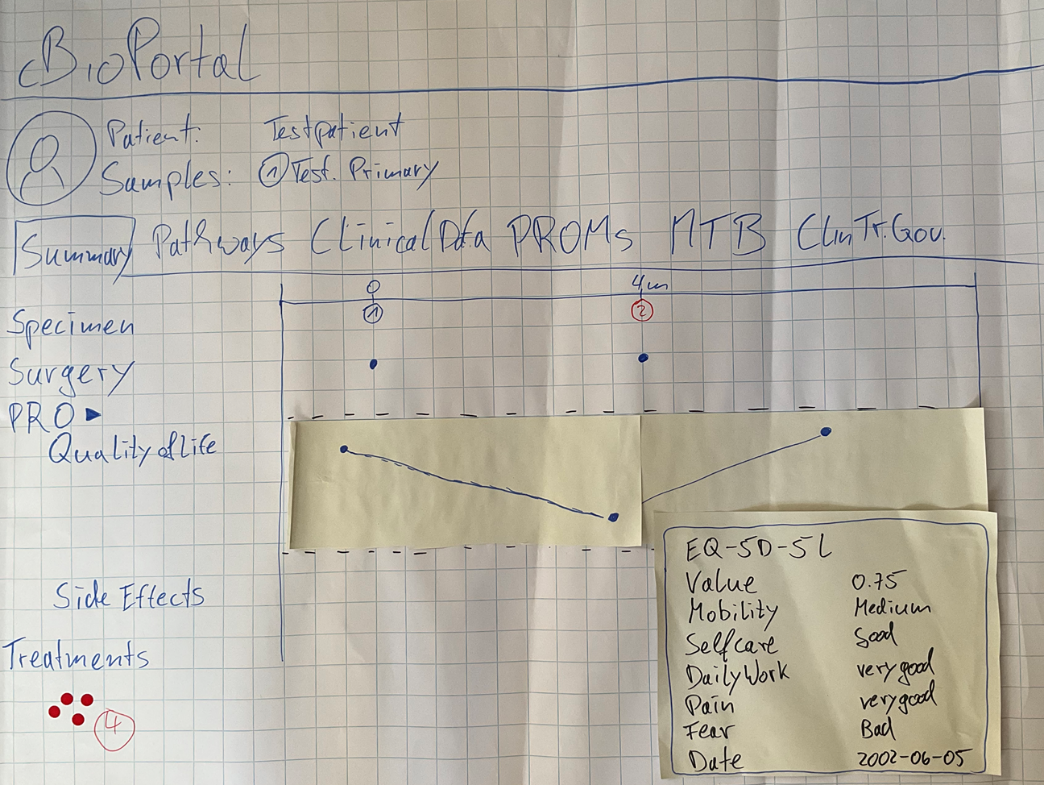
**

Figure 5: Prototype of design group 3, summary tab

**
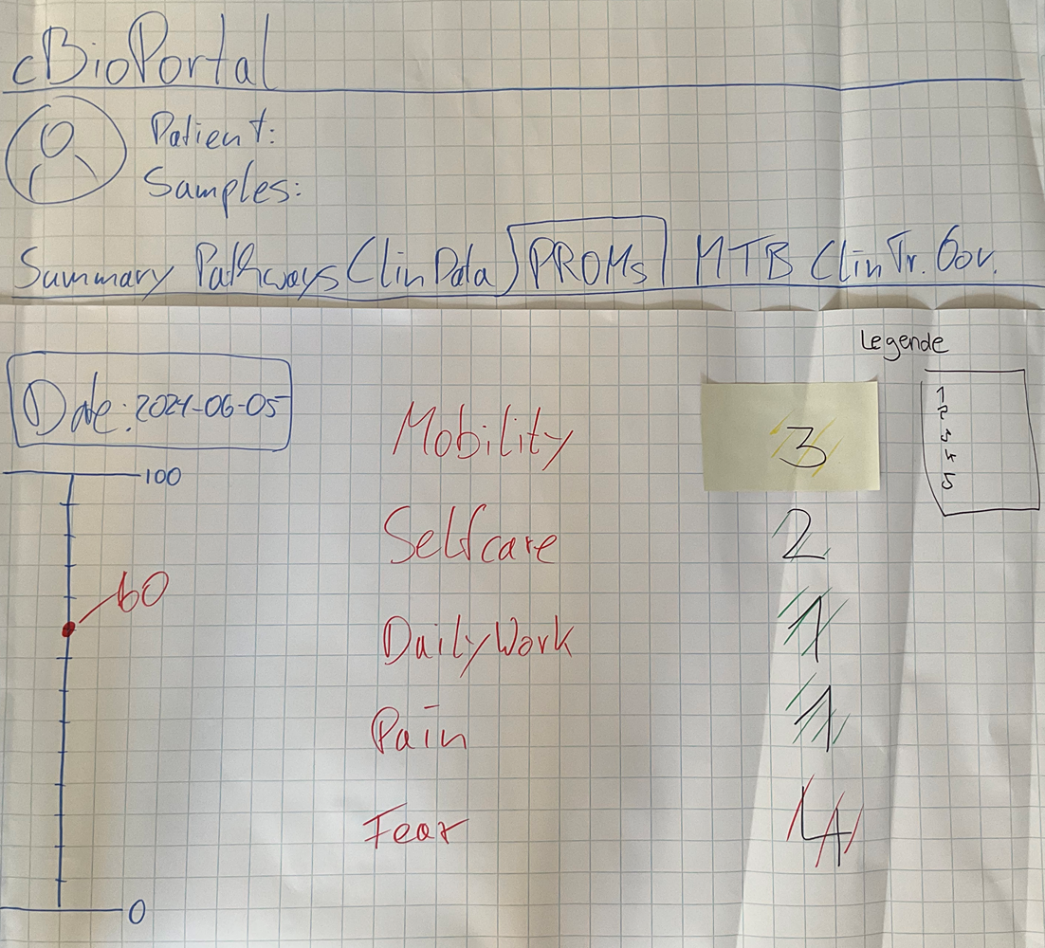
**

Figure 6: Prototype of design group 3, PROMs tab

**
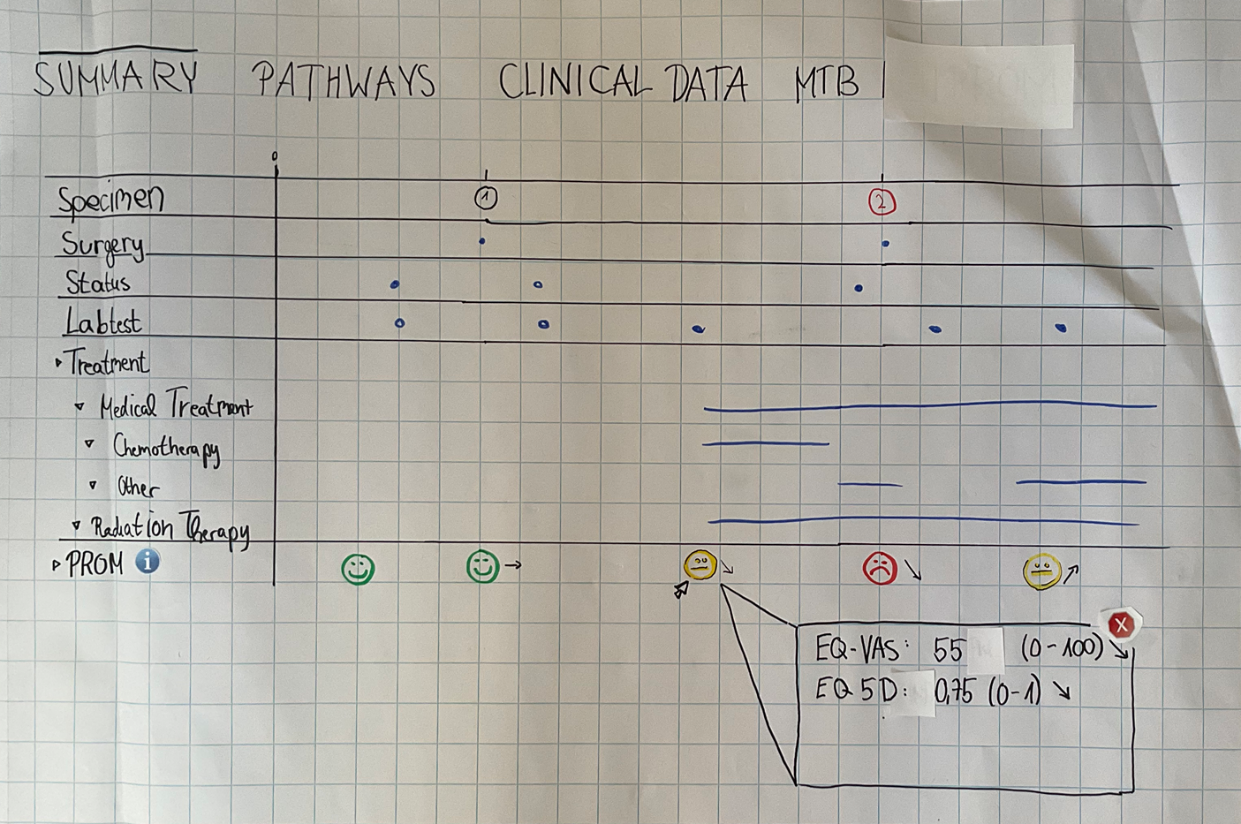
**

Figure 7: Prototype of design group 4, summary tab

**
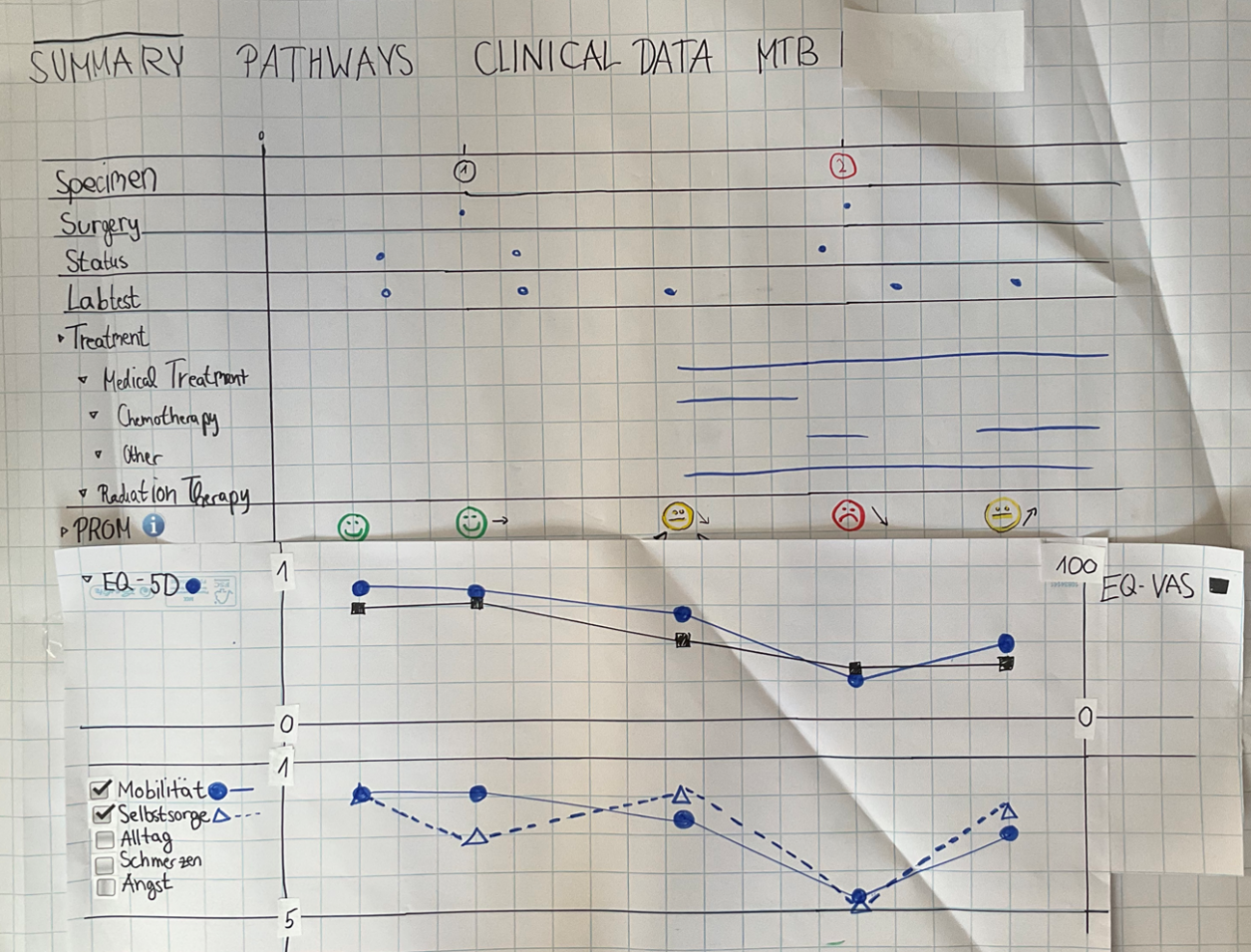
**

Figure 8: Prototype of design group 4, summary tab extended

# Results of the prototype usability evaluation

## Part 1: Description of participants

| **Item** | **Response** | **Count** | **Percentage** |
| --- | --- | --- | --- |
| **Institution** |  |  |  |
|  | Mk1 | 1 | 10 |
|  | NCT/ UCC | 1 | 10 |
|  | MHH | 1 | 10 |
|  | Tu München | 1 | 10 |
|  | University Hospital Dresden | 3 | 30 |
|  | Charité Berlin | 1 | 10 |
|  | No response | 2 | 20 |
| **Specialty** |  |  |  |
|  | Pathology | 0 | 0 |
|  | Hematology / Oncology | 7 | 70 |
|  | Human Genetics | 1 | 10 |
|  | Systems Medicine | 0 | 0 |
|  | Bioinformatics | 0 | 0 |
|  | Epidemiology | 1 | 10 |
|  | No response | 1 | 10 |
| **Gender** |  |  |  |
|  | Female | 2 | 20 |
|  | Male | 7 | 70 |
|  | Diverse | 0 | 0 |
|  | No response | 1 | 10 |
| **MTB Membership** | |  |  |
|  | Yes | 8 | 80 |
|  | No | 1 | 10 |
|  | No response | 1 | 10 |
| **Prior experience with cBioPortal** | | |  |
|  | None | 1 | 10 |
|  | Low | 5 | 50 |
|  | Medium | 2 | 20 |
|  | High | 1 | 10 |
|  | No response | 1 | 10 |

| **Item** | **Mean** | **SD** |
| --- | --- | --- |
| **Professional experience** | 11.89 | 6.76 |
| **Age** | 42.78 | 7.69 |

## Part 2: Evaluation of the prototype

| **Item** | **Response** | | | | | |
| --- | --- | --- | --- | --- | --- | --- |
| **SUS Score** | Strongly disagree | Somewhat disagree | Neutral | Somewhat agree | Strongly agree | No response |
| I can imagine using the application regularly. | 1 | 1 | 1 | 6 | 1 | 0 |
| I find the application unnecessarily complex. | 1 | 3 | 6 | 0 | 0 | 0 |
| I find the application easy to use. | 0 | 1 | 4 | 3 | 2 | 0 |
| I think I would need technical support to use the application. | 5 | 1 | 3 | 1 | 0 | 0 |
| I find that the various functions of the application are well integrated. | 1 | 1 | 2 | 4 | 2 | 0 |
| I find there are too many inconsistencies in the application. | 2 | 3 | 4 | 1 | 0 | 0 |
| I can imagine that most people would learn to master the application quickly. | 0 | 2 | 2 | 3 | 3 | 0 |
| I find the operation very cumbersome. | 3 | 4 | 2 | 1 | 0 | 0 |
| I would feel very confident using the application. | 0 | 0 | 5 | 3 | 2 | 0 |
| I would need to learn a lot of things before I could work with the application. | 3 | 3 | 3 | 1 | 0 | 0 |
| **Intuitiveness** | Not at all intuitive | Less intuitive | Neutral | Rather intuitive | Very intuitive | No response |
| Integration of EQ-5D-5L survey values in timeline in Summary tab | 0 | 0 | 4 | 5 | 1 | 0 |
| Display of current EQ score in PROMs tab | 0 | 1 | 3 | 3 | 3 | 0 |
| Line diagram with EQ-5D-5L total scores | 0 | 1 | 3 | 2 | 4 | 0 |
| Line diagram with EQ-5D-5L detailed view | 0 | 1 | 3 | 4 | 2 | 0 |
| **Usefulness** | Not at all useful | Less useful | Neutral | Rather useful | Very useful | No response |
|  | 0 | 2 | 4 | 3 | 1 | 0 |
| **Completeness** | Very incomplete | Rather incomplete | Partly complete | Rather complete | Very complete | No response |
|  | 0 | 0 | 2 | 8 | 0 | 0 |
| **Visual Design** | Not at all helpful | Less helpful | Neutral | Rather helpful | Very helpful | No response |
| Integration of EQ-5D-5L survey values in timeline in Summary tab | 0 | 1 | 4 | 3 | 2 | 0 |
| Display of current EQ score in PROMs tab | 0 | 0 | 4 | 3 | 3 | 0 |
| Line diagram with EQ-5D-5L total scores | 0 | 1 | 4 | 3 | 2 | 0 |
| Line diagram with EQ-5D-5L detailed view | 0 | 2 | 3 | 3 | 2 | 0 |
| **Interactive elements** | Not at all helpful | Less helpful | Neutral | Rather helpful | Very helpful | No response |
| Mouseover | 0 | 0 | 3 | 6 | 1 | 0 |
| ON/OFF switches | 0 | 0 | 3 | 5 | 2 | 0 |
| **Overall Visual Design** | Very dissatisfied | Rather dissatisfied | Partly satisfied | Rather satisfied | Very satisfied | No response |
|  | 1 | 0 | 4 | 4 | 1 | 0 |

| **Item** | **Response** | **Count** | **Percentage** |
| --- | --- | --- | --- |
| **Missing** |  |  |  |
|  | Warning signals | 5 | 50 |
|  | Coloured markings | 1 | 10 |
|  | Comparison scales | 5 | 50 |
|  | Customisation options | 0 | 0 |
|  | Integration with molecular or clinical data | 3 | 30 |
|  | Other | 1 | 10 |
|  | None | 1 | 10 |
| **Unnecessary graphics** | |  |  |
|  | Yes | 1 | 10 |
|  | No | 7 | 70 |
|  | Not specified | 2 | 20 |

**Additional or alternative forms of visualization:**

- Bar charts (1 mention)
- Trend lines for comparison with healthy patients and normal range/normal values (2 mentions)
- Clear labeling of axes (e.g., what value 1 or 5 means) (1 mention)
- Direct display in the overview without mouseover (1 mention)

**Suggestions for improvement**

- The EQ-5D is not tumor-specific, therefore the EORTC QLQ30 should be used as a questionnaire instead. The representations of symptom scores and functionalities would then need to be adjusted to this questionnaire. (2 mentions)
- Display with value and bars easier to grasp, there e.g., good values marked in color as green bars and bad values as red bars. (1 mention)
- Comparison values are missing (although the question arises whether comparison with the German standard makes sense here). However, other PROMs offer such comparison values. T-scores would probably be easiest, which are (after familiarization) quite easy to understand. (1 mention)
- In the overall view, a trend of the PROM results should be displayed. The display as a point with mouseover is not meaningful and having to open the "PROMs" tab individually each time is perceived as cumbersome. (1 mention)

## Part 3: Evaluation of the Usefulness of PROMs

| **Item** | **Response** | | | | | |
| --- | --- | --- | --- | --- | --- | --- |
| Clinical Workflow Impact | Very negative | Rather negative | Neutral | Rather positive | Very positive | No response |
|  | 0 | 0 | 7 | 1 | 2 | 0 |
| PROMs Integration Usefulness | Not at all useful | Rather not useful | Neutral | Rather useful | Very useful | No response |
|  | 1 | 1 | 3 | 3 | 2 | 0 |
| Added Value | Very low | Rather low | Medium | Rather high | Very high | No response |
|  | 2 | 3 | 3 | 1 | 1 | 0 |

# References

[1] E. A. C. Albers *et al.*, *Visualization formats of patient-reported outcome measures in clinical practice: a systematic review about preferences and interpretation accuracy*, vol. 6, no. 1. Springer International Publishing, 2022. doi: 10.1186/s41687-022-00424-3.

[2] S. L. Hancock *et al.*, “Feedback of patient-reported outcomes to healthcare professionals for comparing health service performance: A scoping review,” *BMJ Open*, vol. 10, no. 11, 2020, doi: 10.1136/bmjopen-2020-038190.

[3] M. Brundage, B. Bass, R. Jolie, and K. Foley, “A knowledge translation challenge: Clinical use of quality of life data from cancer clinical trials,” *Quality of Life Research*, vol. 20, no. 7, pp. 979–985, 2011, doi: 10.1007/s11136-011-9848-0.

[4] K. C. Smith *et al.*, “Engaging stakeholders to improve presentation of patient-reported outcomes data in clinical practice,” *Supportive Care in Cancer*, vol. 24, no. 10, pp. 4149–4157, 2016, doi: 10.1007/s00520-016-3240-0.

[5] L. H. Liu *et al.*, “Patient and clinician perspectives on a patient-facing dashboard that visualizes patient reported outcomes in rheumatoid arthritis,” *Health Expectations*, vol. 23, no. 4, pp. 846–859, 2020, doi: 10.1111/hex.13057.

[6] E. Tolbert, M. Brundage, E. Bantug, A. L. Blackford, K. Smith, and C. Snyder, “Picture This: Presenting Longitudinal Patient-Reported Outcome Research Study Results to Patients,” *Medical Decision Making*, vol. 38, no. 8, pp. 994–1005, 2018, doi: 10.1177/0272989X18791177.

[7] C. F. Snyder *et al.*, “What do these scores mean? Presenting patient-reported outcomes data to patients and clinicians to improve interpretability,” *Cancer*, vol. 123, no. 10, pp. 1848–1859, 2017, doi: 10.1002/cncr.30530.

[8] M. Brundage *et al.*, “Presenting comparative study PRO results to clinicians and researchers: beyond the eye of the beholder,” *Quality of Life Research*, vol. 27, no. 1, pp. 75–90, 2018, doi: 10.1007/s11136-017-1710-6.

[9] D. Ragouzeos *et al.*, “‘Am I OK?’ using human centered design to empower rheumatoid arthritis patients through patient reported outcomes,” *Patient Educ Couns*, vol. 102, no. 3, pp. 503–510, 2019, doi: 10.1016/j.pec.2018.10.016.

[10] R. Jagsi *et al.*, “Qualitative analysis of practicing oncologists’ attitudes and experiences regarding collection of patient-reported outcomes,” *J Oncol Pract*, vol. 9, no. 6, 2013, doi: 10.1200/JOP.2012.000823.

[11] A. L. Hartzler, S. Chaudhuri, B. C. Fey, D. R. Flum, and D. Lavallee, “Integrating Patient-Reported Outcomes into Spine Surgical Care through Visual Dashboards: Lessons Learned from Human-Centered Design,” *eGEMs (Generating Evidence & Methods to improve patient outcomes)*, vol. 3, no. 2, p. 2, 2015, doi: 10.13063/2327-9214.1133.

[12] W. Kuijpers *et al.*, “Patients’ and health professionals’ understanding of and preferences for graphical presentation styles for individual-level EORTC QLQ-C30 scores,” *Quality of Life Research*, vol. 25, no. 3, pp. 595–604, 2016, doi: 10.1007/s11136-015-1107-3.

[13] L. Watson *et al.*, “Utilizing Patient Reported Outcome Measures (PROMs) in ambulatory oncology in Alberta: Digital reporting at the micro, meso and macro level,” *J Patient Rep Outcomes*, vol. 5, no. 2, pp. 1–9, 2021, doi: 10.1186/s41687-021-00373-3.

[14] O. C. Damman *et al.*, “Using PROMs during routine medical consultations: The perspectives of people with Parkinson’s disease and their health professionals,” *Health Expectations*, vol. 22, no. 5, pp. 939–951, 2019, doi: 10.1111/hex.12899.

[15] M. D. Brundage, K. C. Smith, E. A. Little, E. T. Bantug, and C. F. Snyder, “Communicating patient-reported outcome scores using graphic formats: results from a mixed-methods evaluation,” *Quality of Life Research*, vol. 24, no. 10, pp. 2457–2472, 2015, doi: 10.1007/s11136-015-0974-y.

[16] E. Tolbert, M. Brundage, E. Bantug, A. L. Blackford, K. Smith, and C. Snyder, “In proportion: approaches for displaying patient-reported outcome research study results as percentages responding to treatment,” *Quality of Life Research*, vol. 28, no. 3, pp. 609–620, 2019, doi: 10.1007/s11136-018-2065-3.

[17] J. Izard, A. Hartzler, D. I. Avery, C. Shih, B. L. Dalkin, and J. L. Gore, “User-centered design of quality of life reports for clinical care of patients with prostate cancer,” *Surgery (United States)*, vol. 155, no. 5, pp. 789–796, 2014, doi: 10.1016/j.surg.2013.12.007.

[18] A. L. Hartzler, J. P. Izard, B. L. Dalkin, S. P. Mikles, and J. L. Gore, “Design and feasibility of integrating personalized PRO dashboards into prostate cancer care,” *Journal of the American Medical Informatics Association*, vol. 23, no. 1, pp. 38–47, 2016, doi: 10.1093/jamia/ocv101.
